# The William Pepper Memorial Volume

**Published:** 1900-11

**Authors:** 


**A Monthly Review of Medicine and Surgery.**

*EDITOR:*

WILLIAM WARREN POTTER, M. D.

All communications, whether of a literary or business nature, books for review and exchanges should be addressed to the editor: 284 FRANKLIN STREET, BUFFALO, N. Y.

VOL. XL.—LVI. NOVEMBER, 1900. NO. 4.

### THE WILLIAM PEPPER MEMORIAL VOLUME.

A MEMORIAL volume dedicated to the memory of the late Dr. William Pepper has recently been published by some of his former students, under the leadership of Dr. Alfred Stengel, of Philadelphia. It is a noble act reflecting credit and honor alike on the William Pepper Laboratory of Clinical Medicine and on the Phœbe A. Hearst foundation, at the same time perpetuating the name and fame of Dr. Pepper in one more special way.

Dr. William Pepper was a many-sided man and in no position did he excel more than in that of his chosen field surrounded by bright, active, energetic young men. He was a steadfast friend to the younger element of the profession, and this volume testifies to the esteem in which he was held by these same active workers, associated with their master in the investigations conducted at the William Pepper Laboratory of Clinical Medicine. No better memorial could be erected to their chief, than this volume of original research, which reflects so much credit on all participating in its publication.

Very appropriate is the beautiful frontispiece, a steel engraving of Dr. Pepper, with a copy of his autograph underneath, printed on heavy plate paper. The likeness is an excellent one and was taken a short time before the doctor's death.

The contributors have chosen subjects in which they have shown superior excellence and portrays the character of work done at this laboratory. The table of contents embraces the following papers: Two cases of muscular dystrophy with necropsy; and a case of amyotrophic lateral sclerosis, in which degeneration was traced from the cerebral cortex to the muscles, by William G. Spiller, M.D.

A contribution to the study of (*a*) iron infiltration in the ganglion cells; (*b*) Forced movements due to cellular degeneration of the cerebellum following rattlesnake poisoning, by D. J. McCarthy, M.D.

A fatal case of sulphonal poisoning, by Alonzo Englebert Taylor, M.D., and Joseph Sailer, M.D.

Melanotic sarcoma of the spinal cord, by Joseph Sailer, M.D.

Studies in leukemia, by Alonzo Englebert Taylor, M.D.

On the pathology of the erythrocyte, by Alfred Stengel, M.D.

The Restitution of the blood plasm following intravenous saline injections after hemorrhage, by A. E. Taylor, M.D., and C. H. Frazier, M.D.

The influence of immoderate water drinking upon metabolism and absorption, by D. L. Edsall, M.D.

An experimental study of the etiology of appendicitis, by Charles H. Frazier, M.D.

Primary endothelioma of the left superior pulmonary vein, by Joseph Sailer, M.D.

A clinical method for the estimation of breast milk proteids, by George Woodward, M.D.

The etiology of pertussis—the bacillus of Czaplewski-Hensel by Joseph Walsh.

A list of the previous contributions from the laboratory completes this volume of 479 pages, every one of which is valuable and instructive.

Some of the articles are handsomely and profusely illustrated, notably those by Dr. Spiller, Dr. Sailer, and that on experimental appendicitis, by Dr. Frazier.

The director of the laboratory, Dr. Alfred Stengel, deserves much credit for the excellent care shown in the selection and character of articles and for the general appearance and typography of the volume.

